# Selection of Reference Genes and *HSP17.9A* Expression Profiling in Heat-Stressed Grapevine Varieties

**DOI:** 10.3390/genes15101283

**Published:** 2024-09-30

**Authors:** Ana Carvalho, Christina Crisóstomo, Fernanda Leal, José Lima-Brito

**Affiliations:** 1 Plant Cytogenomics Laboratory, University of Trás-os-Montes and Alto Douro (UTAD), Laboratorial Complex, Room A1.09, Quinta de Prados, 5000-801 Vila Real, Portugal; christina98@live.com.pt (C.C.); jbrito@utad.pt (J.L.-B.); 2 Department of Genetics and Biotechnology, University of Trás-os-Montes and Alto Douro (UTAD), Laboratorial Complex, Quinta de Prados, 5000-801 Vila Real, Portugal; fleal@utad.pt; 3 Centre for the Research and Technology of Agro-Environmental and Biological Sciences (CITAB), University of Trás-os-Montes and Alto Douro (UTAD), Quinta de Prados, 5000-801 Vila Real, Portugal; 4 Institute for Innovation, Capacity Building and Sustainability of Agri-Food Production (Inov4Agro), University of Trás-os-Montes and Alto Douro (UTAD), Quinta de Prados, 5000-801 Vila Real, Portugal

**Keywords:** gene expression, heat shock protein, in vitro culture, quantitative real-time PCR (qPCR), *Vacuolar ATPase subunit G* (*VAG*) gene, *Ubiquitin-conjugating enzyme* (*UBC*) gene

## Abstract

Background: “Touriga Franca” (TF) and “Touriga Nacional” (TN) are grapevine varieties cultivated in the ‘Douro Superior’ subregion (Northern Portugal) that experience stressful environmental conditions during the summer. Objectives: Aiming to profile the expression of stress-responsive genes by quantitative real-time PCR (qPCR) in TF and TN plants growing naturally, three candidate reference genes were first tested under controlled conditions. Methods: To simulate a summer’s day, TF and TN in vitro plants were exposed to 32 °C–3 h (heat acclimation) and 42 °C–1 h (severe heat stress, HS) followed by two recovery periods (32 °C–3 h and 24 °C–24 h). Leaf samples were collected at the end of each phase. Control plants were kept at 24 °C. Results: Among the candidate reference genes, the *UBC* and *VAG* pair showed the highest stability. The suitability of these genes for qPCR was validated by *heat shock protein 17.9A* (*HSP17.9A*) gene profiling. The *HSP17.9A* expression was up-regulated in both varieties and all experimental phases except in TF control plants. TN showed the highest *HSP17.9A* relative expression ratio after severe HS. Conclusions: TN responded faster than TF to the induced heat shocks. The *UBC*, *VAG*, and *HSP17.9A* genes revealed to be suitable for further qPCR assays in TF and TN grapevine varieties.

## 1. Introduction

Temperature is one of the abiotic factors crucial for plant growth, development, and yield, but its increase above the physiological optimum results in heat stress (HS) affecting the regularity of these biological processes [1]. The occurrence of HS, currently aggravated by global warming, constrains the flowering and fruit development and strongly reduces the yield in several crops [1].

*Vitis vinifera* L. (grapevine) is a highly economically important fruit species that is cultivated worldwide. Considering the threats of climate change to the grapevine productivity, yield, berry, and wine quality [2,3,4], the use of biotechnological and molecular approaches for the evaluation of the success of adaptation or management strategies or characterisation of genetic resources aiming the selection of more tolerant genotypes targeting their use and/or improvement is demanded. Plant growth under greenhouse conditions, hydroponics, and in vitro culture are controlled experimental systems that have been used to study the grapevine responses to various specific stresses [5,6,7,8,9,10]. The results of stress studies performed under controlled environments cannot be easily extrapolated to natural conditions [5] due to the interaction of multiple stresses occurring at any given moment in the field [11]. Nevertheless, under controlled conditions, plant responses to the induced stress can be exacerbated further. Therefore, the genotypes selected as more resilient in these stress studies will certainly withstand extreme abiotic factors in natural environment and/or have the potential to be genetically improved [8].

More than 250 Portuguese grapevine varieties, including the red wine “Touriga Franca” (TF) and “Touriga Nacional” (TN), are officially recommended for wine production [12]. Due to their oenological traits, TF and TN are considered top varieties for the Portuguese wine industry [3]. These two red wine varieties are suitable for the production of wines with the protected designation of origin ‘Douro’ and ‘Porto’ [13]. TF and TN are widely cultivated in the ‘Douro Demarcated Region’ (DDR), which integrates the World Heritage List of UNESCO and constitutes the Portuguese region with the largest wine production [3]. The TF and TN varieties produce high-quality wines, and some of those have been internationally awarded. Despite the high adaptation of these two varieties to the DDR microclimate, currently, their cultivation is spread by different Portuguese regions, including the Azores archipelago [14,15]. TN has excellent agronomic performance, high vigour, fertility, and medium-to-high yield [14]. TF presents medium-to-high yield, low-to-medium fertility, and regular productivity [15]. In terms of resilience, TF is pointed out as tolerant to abiotic factors and resistant to pests and diseases [15]. As previously reported, TN is susceptible to water stress, but it can withstand temperatures up to 40 °C (HS) as long as water is plenty [5,8,16,17]. The same should be valid for TF once they share the extreme environmental growing conditions of the ‘Douro Superior’ subregion and remaining DDR [3,14,15,18,19,20]. Nevertheless, and probably due to its broader cultivation in Portugal [14,15], TN has been the target of more stress studies than TF [8,16,17,21,22]. Despite the combination of stressful abiotic factors simultaneously occurring in the field during consecutive summer days, the high temperature is a primary concern for wine producers since it negatively impacts the wine’s quality [5,16,21]. To mitigate the negative consequences of the summer’s stressful conditions, wine producers have chosen different grapevine varieties or treating plants with foliar protective compounds such as phytohormones or kaolin [16,20,21]. The exogenous application of phytohormones, before or during HS, can potentially mitigate the induced damage and improve the thermotolerance of the treated plants, avoiding the reduction in productivity and yield, which is particularly important in agricultural crops [23]. The foliar spraying with kaolin decreases the temperature at the leaf’s surface [20,21].

The evaluation of the success of short-term adaptation measures can be assessed by quantitative real-time PCR (qPCR) focused on the expression profiling of abiotic stress-responsive genes in treated and untreated grapevine plants growing naturally. However, the realisation of such molecular approaches should be preceded by selecting the best candidate reference (housekeeping) genes and their validation. To avoid the loss of essential leaf samples collected in the field, previous analyses can be performed under controlled conditions using in vitro-grown plants of the same grapevine varieties.

Plants evolved adaptive mechanisms at the cellular, physiological, biochemical, and molecular levels to deal with adverse environmental conditions. The coordination of these processes results from the activation of various genes and epigenetic changes in response to high temperature [4,20,22,24]. In response to heat, plants activate (i) biosynthetic pathways of phytohormones; (ii) the synthesis of antioxidant enzymes; (iii) phytochromes and physical changes in lipid membranes; (iv) the induction of heat shock transcription factors (HSFs) whose targets are heat shock proteins (HSPs) and reactive oxygen species (ROS)—scavenging enzymes; (v) the expression of transposable elements; and (vi) genes encoding HSPs whose accumulation prevents irreversible damage on proteins and confers thermotolerance [20,21,22,23,24,25].

The HSPs are coded by different gene families [26]. Among the HSPs, the small heat shock proteins (sHSPs), with a molecular weight of 12–40 kDa, are prevalent in plants and differ from HSPs in their response to stress [23,26]. The accumulation of sHSPs in plants confers thermotolerance [23,26]. Under high temperatures, the sHSPs prevent the clumping and assist in the folding of numerous proteins in different species [23,26].

This work aimed to select candidate reference genes suitable for qPCR assays in TF and TN in vitro-grown plants and their validation based on the expression profiling of the *HSP17.9A* gene (encoding for the small HSP17.9A protein) during an experimental setup that intends to simulate a summer’s day in the ‘Douro Superior’ subregion. Attempting to mimic the temperature fluctuations throughout a summer’s day, the in vitro-grown TF and TN plants were exposed to heat acclimation (32 °C–3 h, moderate HS); extreme HS (42 °C–1 h) to simulate the solar noon; followed by two recovery periods, a shorter one at 32 °C–3 h and a longer one for 24 h at 24 °C, which simulate the temperature reduction throughout the evening and night, respectively. The selected candidate reference gene(s) will be used in transcriptional studies in TF and TN plants naturally growing under stressful summer conditions.

## 2. Materials and Methods

### 2.1. Plant Material and HS Induction

In vitro-grown plants that were 11 months old, from the two red-wine-producing varieties ‘Touriga Franca’ (TF) and ‘Touriga Nacional’ (TN), were used for HS induction. These plants grew on a semisolid MS basal medium [27], pH 5.6, supplied with 2% sucrose, without phytohormones, at 24 °C (±1 °C), under a photoperiod of 16 h and light intensity of 300 μmol m^−2^ s^−1^, within a growth chamber Fitoclima ‘Walk-in’—model 20000E (Aralab). Per grapevine variety, six plants with 12 cm height and 8–10 fully expanded leaves were used. The plants were maintained at 24 °C (±1 °C) until the beginning of the HS induction and recovery experimental setup, which was applied to three plants per grapevine variety, consisting of four consecutive steps: (i) acclimation period for 3 h at 32 °C (moderate HS); (ii) severe HS for 1 h at 42 °C; (iii) 3 h at 32 °C (first recovery period); and (iv) 24 h at 24 °C (more extensive recovery period). Three plants from each variety were kept within the growth chamber at 24 °C (±1 °C) to be used as the control group. At the end of each stress or recovery step, leaf samples of each grapevine variety were collected within the flow chamber, immediately frozen in liquid nitrogen and kept at −80 °C until total RNA isolation.

### 2.2. Extraction of Total RNA, cDNA Synthesis, and qPCR Assays

The total RNA extraction, reverse transcription, qPCR primer information, and experimental design followed the minimum information for publication of quantitative real-time PCR experiments (MIQE) guidelines [28].

The frozen (−80 °C) grapevine leaves collected at the end of each experimental phase were grounded in liquid nitrogen for total RNA extraction using a CTAB-based protocol [29]. RNA integrity was evaluated after electrophoresis on 2% agarose gels under denaturing conditions as previously described [29]. The total RNA samples were purified with *DNase* I using a PureLink^TM^ RNA Mini Kit (Ambion^®^, Life Technologies^TM^, Invitrogen, Carlsbad, CA, USA) following the manufacturer’s instructions. RNA purity and quantification were assessed by spectrophotometry using Nanodrop^TM^ ND-1000 (Thermo Fisher Scientific, Inc., Waltham, MA, USA) equipment. For the complementary DNA (cDNA) synthesis, an amount of 200 ng of total RNA and a High-Capacity cDNA Reverse Transcription kit (Applied Biosystems, Foster City, CA, USA) were used. For the qPCR assays, individual cDNA samples were diluted with ultra-pure DNase/RNase-free distilled water to 40 ng µL^−1^.

The qPCR primers (Table 1) were specifically designed for *V. vinifera* by other authors [23,30], and their synthesis were ordered from STAB Vida, Lda., FCT/UNL (Caparica, Portugal).

All qPCR primers were first checked by standard PCR using cDNA samples as templates, followed by electrophoresis on 2% agarose gels stained with ethidium bromide. Standard curves based on 10× dilution series of the pooled cDNA samples of all plant materials were performed for the determination of amplification efficiency (E) and correlation coefficient (r) per gene using Equation (1).

[E = 10^(−1/slope)^ − 1] (1)

The qPCR assays were performed in 96-well Bio-Rad^®^ Multiplate PCR Plates 96-well clear (Cat# MLP9601; Bio-Rad Laboratories, Inc., Hercules, CA, USA), sealed with Microseal^®^ ‘B’ Adhesive Seals for PCR Plates (Bio-Rad^®^; Cat.# MSB1001), using Stratagene Mx3005P qPCR (Agilent Technologies^®^, Santa Clara, CA, USA) equipment. The qPCR reaction mixture and amplification conditions, including the production of denaturing (melting) curves for each amplicon, were the same as described earlier [20]. Three technical and biological replicates (n = 3) representing three different plants per grapevine variety, gene, and experimental phase were performed. Negative controls for each gene were included per plate. The two nearest quantification cycle (Cq) values of the biological and technical replicates carried out per reference and target gene were used to determine the mean Cq values per ‘grapevine variety × experimental phase’ interaction (Figure S1). The average Cq values were normalised to the geometric mean of the Cq values of the selected reference gene(s) resulting in the mean ∆Cq values presented in Figure S1.

The relative expression ratio of the target *HSP17.9A* gene was determined with Equation (2) [31].

(2) $$\mathrm{Relative}\;\mathrm{expression}\;\mathrm{ratio}=\frac{{\left(E\;\mathrm{target}\right)}^{∆\mathrm{target}\;\mathrm{ex}\left(\mathrm{control}-\mathrm{sample}\right)}}{{\left(E\;\mathrm{reference}\right)}^{∆\mathrm{referenceamp}\left(\mathrm{control}-\mathrm{sample}\right)}}$$

In Equation (2), the E target represents the efficiency amplification value of the *HSP17.9A* gene. The E reference consists of the geometric mean of the efficiency values of the two selected reference genes. ∆Cq represents the difference between the Cq values achieved in the control and treated plants for the *HSP17.9A* (target gene) and the geometric mean of the Cq values of the two selected reference genes. Values of relative expression ratio above 1 indicate up-regulation, whereas values between 0 and 1 were considered down-regulation [32].

### 2.3. Evaluation and Selection of the Reference Genes for qPCR Assays

For selecting the best pair of reference genes, among the three tested candidates, *Phosphoenolpyruvate carboxylase* (*PEG*), *Vacuolar ATPase subunit G (VAG)*, and *Ubiquitin-conjugating enzyme (UBC)*, the methods GeNorm [33], Normfinder [34], Bestkeeper [35], and Delta-CT [36] were used. The geometric mean of the standard deviation (S.D.) values per candidate gene achieved with the different methods were considered for comprehensive ranking.

### 2.4. Statistical Analyses

The expression ratio of the *HSP17.9A* target gene was determined in relation to the control plants (kept at 24 °C) per ‘grapevine variety × experimental phase’ interaction. The relative expression values of the *HSP17.9A* gene were calculated based on the mean Cq values of two biological and technical replicates (n = 2), whose standard deviation was lower than 0.5, using the Livak and Schmittgen [32] method. To evaluate the statistical significance among these values of relative expression ratio, the equality of variances F test and the one-sample sign test hypothesised variance were used. To increase the robustness of the statistical analysis of the expression data, the values of relative expression determined per replicate of each ‘grapevine variety × experimental phase’ interaction were subjected to one-way analysis of variance (ANOVA), the post hoc Fisher’s protected least significant difference (PLSD) test, and the equality of variances F test. All the previous statistical tests were performed with the software Statview 5.0 (SAS Institute, Inc., Copyright © 1992–1998, Cary, NC, USA). The *p*-value significance of these statistical analyses was set for probabilities lower than 5% (*p* < 0.05) and 0.1% (*p* < 0.001). In addition, the expression ratio values of the replicates per grapevine variety × experimental phase’ interaction were used for the calculation of mean and standard deviation values for further determination of the confidence interval (C.I.) using an α value of 0.95 using Microsoft^®^ Excel^®^ 2010.

## 3. Results

**Figure 1:**
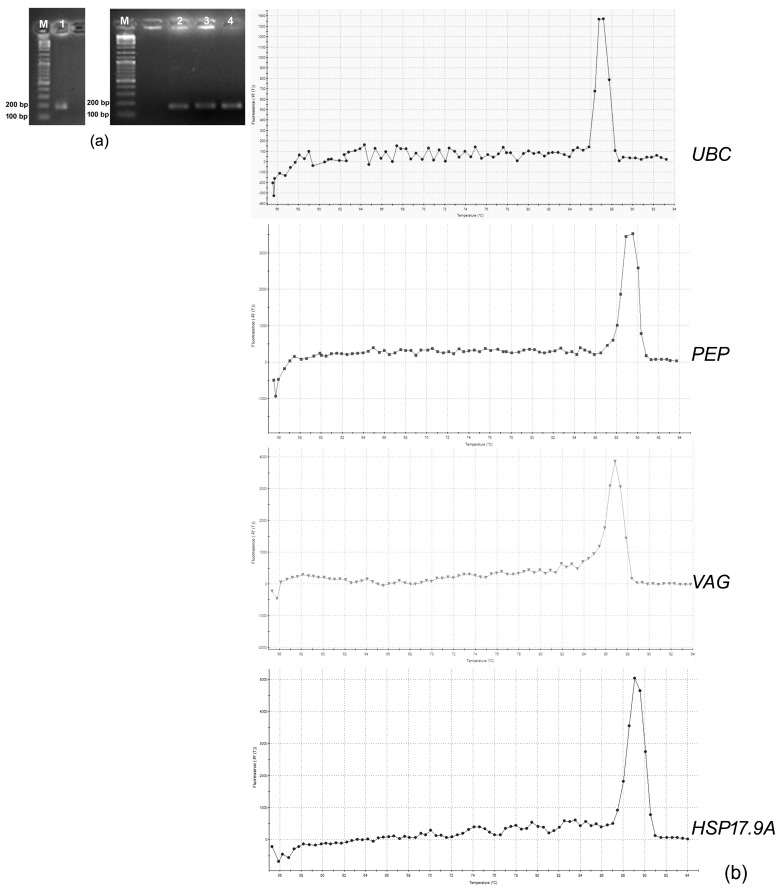
(**a**) Amplified products of the candidate reference genes (lane 1—*PEP*; lane 2—*UBC*, and lane 3—*VAG*) and of the target gene *HSP17.9A* (lane 4) visualised after electrophoresis on 2% agarose gels. In each gel, the molecular weight marker GeneRuler^TM^ 100 bp DNA Ladder Plus (#SM0321, Thermo Fisher Scientific Baltics UAB, Vilnius, Lithuania) was loaded. (**b**) Dissociation curves of the candidate reference genes and the *HSP17.9A* target gene (identified in the image).

After electrophoresis on agarose gels, the specificity of the primers and the expected amplicon size of each gene were confirmed (Figure 1a). The evaluation of the dissociation curves produced for each gene also revealed the amplicons’ specificity (Figure 1b).

### 3.1. Selection of the Candidate Reference Genes

For the selection of the best candidate reference gene(s), the Cq values and/or the log2-transformed expression ratio of the *PEP*, *VAG*, and *UBC* genes were evaluated individually and in pairwise analyses of gene expression variation using different methods (Table 2).

The mean Cq values achieved with the candidate reference genes *PEP*, *VAG*, and *UBC* were 33.43, 22.78, and 20.22, respectively. The lowest mean Cq value presented by the *PEP* gene indicates lower expression than that observed for the *UBC* and *VAG* genes. Regardless of the method used for gene stability ranking, in both individual and pairwise analyses, the *UBC* and *VAG* genes showed the lowest S.D. values, as well as the lowest %C.V. values in Bestkeeper (Table 2). This latter method excludes genes with S.D. values higher than 1.0 and considers them as inconsistent [35], which was the case of *PEP* (Table 2). In NormFinder analysis, the candidate genes with a stability value close to zero suggest the lowest inter-group variation [34]. Among the tested candidates, the genes *UBC* and *VAG* presented the lowest stability values (Table 2). The M-value determined by the GeNorm algorithm should be higher than 0.5 and lower than 1.5 [33]. Concerning the formulas published by these authors, the pairwise analyses performed for each pair of internal control genes allowed us to determine the lowest M-value (1.391) for the combination of genes *UBC* and *VAG* (Table 2). The comprehensive ranking, based on the geometric mean of the S.D. values generated with each method, revealed the lowest average S.D. values for the *UBC* and *VAG* genes (Table 2). Hence, the results of the analysis of the global results presented in Table 2 suggested *UBC* and *VAG* as the best candidates for reference genes given their highest gene expression stability.

### 3.2. Expression Profiling of the HSP17.9A Target Gene

**Figure 2:**
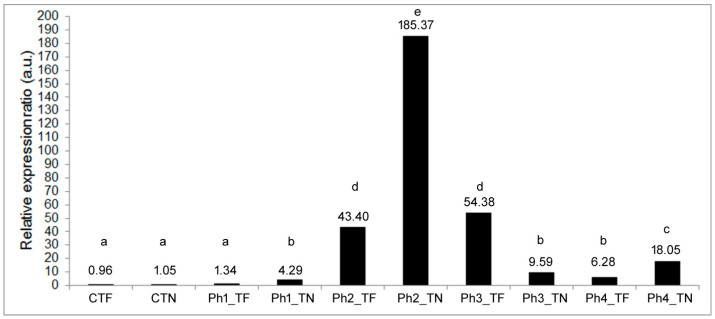
Relative gene expression in arbitrary units (a.u.) of the target gene, *HSP17.9A*, determined for the in vitro TF and TN plants at the end of each experimental phase (Ph1 to Ph4), relative to the control plants. Different lowercase letters among bars represent statistically significant differences (*p* ˂ 0.05). Notes: CTF and CTN—control plants of TF and TN varieties, respectively; Ph1—32 °C for 3 h (heat acclimation); Ph2—42 °C for 1 h (severe HS); Ph3—32 °C for 3 h (first recovery period); and Ph4—24 °C during 24 h (second recovery period).

The *UBC* and *VAG* genes were used for the calculation of the relative expression ratio of the *HSP17.9A* target gene per ‘grapevine variety × experimental phase’ interaction in relation to the control plants that were kept at 24 °C (Figure 2).

As previously reported [32], a relative expression ratio ranging between 0 and 1 is considered down-regulation, whereas values above 1 to infinity constitute up-regulation. The *HSP17.9A* gene showed up-regulation in both varieties and all experimental phases, except for the control TF plants (CTF) (Figure 2).

The slight increase in the *HSP17.9A* expression ratio values shown by the control TN plants (CTN) and TF plants after heat acclimation (Ph1_TF) did not significantly differ (*p* > 0.05) from the CTF expression level (Figure 2). However, a faster up-regulation of the *HSP17.9A* expression was detected in both grapevine varieties after severe HS (Ph2), being more pronounced in TN (Figure 2). This latter variety also responds faster than TF to the temperature reduction as detected at the end of the first recovery period (Ph3) (Figure 2). Nonetheless, at the end of the second and more extensive recovery period (Ph4), the TN variety presented a significantly higher *HSP17.9A* expression ratio than TF, which was approximately 17-fold higher than that in the control plants (CTN) (Figure 2).

Since the relative expression ratio values graphically represented in Figure 2 resulted from calculations involving average relative quantity values, error bars were not exhibited. The statistically significant differences identified among the relative expression values in Figure 2 were estimated with the equality of variances F test and the one-sample sign test hypothesised variance regarding the probability that the difference of expression data among bars was only due to chance. The up-regulation of the *HSP17.9A* gene in most of the cases (Figure 2) refuted this hypothesis, even though, to reinforce the robustness of the expression data analysis, individual values of the relative expression of the target gene were calculated for each replicate and ‘grapevine variety × experimental phase’ interaction and used in additional statistical tests. Table 3 presents the summarised results of the ANOVA, Fisher’s PLSD test, equality of variances F test, and determination of the confidence intervals, which were performed with those values.

The mean values of the relative expression ratio of the *HSP17.9A* gene showed statistically significant differences (*p* ˂ 0.001) among the ‘grapevine variety × experimental phase’ interactions (Table 3), corroborating the results in Figure 2. The C.I. determination for the log fold change values of the *HSP17.9A* gene reinforced the statistically significant differences evidenced by the presented *p*-value (0.001) (Table 3).

Globally, the profiling of the *HSP17.9A* expression indicated a significant and differential relative expression ratio among the ‘grapevine variety × experimental phase’ interactions (*p* ˂ 0.05). Furthermore, these results demonstrated the significant up-regulation of the *HSP17.9A* expression in response to the induced heat shocks, which reached its maximum level at the end of the severe HS (Figure 2).

## 4. Discussion

Considering the high economic importance of viticulture worldwide, it is urgent to evaluate the negative impacts of climate change on vine productivity and wine’s quality. Efforts involving changing management practices and applying short-term adaptation measures have been performed to counteract the negative consequences of stressful environmental conditions in viticulture [20,21,37]. Some of these studies involved the expression profiling of abiotic stress-responsive genes [16,20,21,23,25,30,37]. The realisation of qPCR studies requires the previous screening of candidate reference genes.

Aiming to analyse further the expression of abiotic stress-responsive genes in TF and TN plants naturally growing in the ‘Douro Superior’ subregion under stressful summer conditions [20], previous qPCR assays were performed under a controlled environment using in vitro-grown plants of the same varieties to select and validate suitable reference genes (this work).

The interchange of heat waves and mild temperatures constitutes successive cycles of HS and recovery that can be partially reproduced under controlled conditions. In this work, TF and TN in vitro-grown plants experienced consecutive heat shocks, attempting to simulate the temperature fluctuations occurring during a summer’s day in the ‘Douro Superior’ subregion.

Firstly, three candidate reference genes were tested. Upon the global analysis of different methods, the pair *UBC* + *VAG* was considered suitable for the normalisation of the target gene since these two genes presented the highest expression stability in both grapevine varieties and experimental phases. According to Borges et al. [30], regardless of the abiotic stress, *UBC*, *VAG*, and *PEP* were considered as suitable reference genes for qPCR studies using grapevine leaves as samples. This assumption was previously confirmed [20] and in this study. Borges et al. [30] did not explain why they chose *UBC*, *VAG*, and *PEP* as candidate reference genes. However, these three candidate genes present conserved domains among various plant species and encode for essential enzymes involved in plant growth and development, carbon fixation, cell elongation, and/or maintenance of the cellular redox homeostasis [38,39,40], justifying their constitutive expression. Despite some authors reporting changes in the expression levels of the *UBC* and *VAG* genes in different *Vitis* species and in response to heat stress, those results were achieved in leaf discs and ripened berries, which might constitute responses to wounding and phenological stage, respectively. Hence, the present qPCR results along with those published earlier [20,30] evidenced the suitability of using *UBC* and *VAG* as reference genes for the expression profiling of *HSP17.9A* in *V. vinifera* leaves.

Secondly, the two selected reference genes were validated based on the expression profiling of the target gene, *HSP17.9A*. Plants have evolved gene networks to cope with thermal stress interacting with metabolic and physiological pathways [41]. Heat shock transcription factors (HSFs), HSPs, and other proteins and enzymes are involved in plant HS response [20,41,42]. Although the *HSP17.9A* gene can be expressed in response to various abiotic stresses, its up-regulation under HS has been documented in plants and other organisms [23,26,41]. Therefore, this experience, developed under controlled conditions where only the temperature factor was changed, restricted the results of gene expression to the induced heat shocks. The expression profiling of the *HSP17.9A* gene revealed up-regulation in most of the ‘grapevine variety × experimental phase’ interaction, confirming its significant response to the induced heat shocks. Although this gene can be activated by various abiotic stresses [26], the present results confirmed its up-regulation in response to high temperatures. According to Jung et al. [43], the analysis of gene expression results should include the determination of C.I. for the log fold change since the biological relevance of the differential expression can be more intuitively judged by a fold change than merely by a *p*-value. Furthermore, the differential relative expression ratio was significantly evidenced between the grapevine varieties and among the experimental phases. Also, the high expression ratio of the *HSP17.9A* gene at the end of the second recovery period, mainly in TN, can suggest an enhanced thermotolerance, which has been widely reported in this grapevine variety [8,20,23].

The effectiveness of foliar spraying with kaolin has recently been evaluated in TF and TN plants naturally growing in the ‘Douro Superior’ subregion for two consecutive years, based on cytogenetic, biochemical, and molecular approaches [20]. The latter study included qPCR assays performed with the *UBC* and *VAG* reference genes (selected in this work) and allowed the expression profiling of various target genes, including *HSP17.9A* [20]. As stated earlier, kaolin reduces the temperature at the leaf’s surface [21]. This assumption was confirmed by the down-regulation of the *HSP17.9A* gene in most of kaolin-treated TF and TN plants growing under open-field conditions [20]. These previous results along with the present ones confirmed the suitability of using *HSP17.9A* as a target gene for HS-related transcriptional studies in these two grapevine varieties.

In conclusion, the threats posed by climate change on agriculture increase the pressure and need of developing heat-tolerant crops. Despite the efforts attempting to develop heat-resilient crops through molecular breeding and genetic modification, the knowledge about the mechanisms underlying the plant heat response is still incipient [22,24]. Further in-depth and simultaneous investigations focused on the thermal-responsive hormone signal transduction pathways and their cross-talk, RNA modifications, and use of genomic and machine-learning approaches for identifying cis-regulatory elements associated with heat-stress responsive genes expression have been suggested [22,24]. These investigations should include both biotechnological approaches and field trials pursuing a preliminary characterisation of the HS response under controlled conditions and its further validation in crops growing under natural conditions. As previously demonstrated [6,7] and in this study, the exposure of in vitro-grown grapevine plants to heat shocks can be useful for the characterisation of the HS response. The development of similar approaches in additional grapevine varieties can contribute to the selection of heat-tolerant genotypes.

## Figures and Tables

**Table 1:** qPCR primer information.

Candidate Reference Genes	* NIH—NCBI Reference Sequence	Sequence (5′→3′)	Expected Amplicon Size (bp)	Reference
*Phosphoenolpyruvate* *carboxylase* (*PEP*)	AF236126.1	F: CCTCCTCCTCCAGATTGC R: GGCTTGCTTGATTCCATTATC	198	[30]
*Vacuolar ATPase* *subunit G* (*VAG*)	XM_002281110.1	F: TTGCCTGTGTCTCTTGTTC R: TCAATGCTGCCAGAAGTG	174	[30]
*Ubiquitin-conjugating* *enzyme* (*UBC*)	EE253706 GenBank: EE253706.1	F: CATAAGGGCTATCAGGAGGAC R: TGGCGGTCGGAGTTAGG	161	[30]
**Target gene**	* NIH—NCBI Reference Sequence	**Sequence (5′→3′)**	**Expected amplicon** **size (bp)**	**Reference**
*Class II Heat Shock Protein 17.9A* (*HSP17.9A*)	XM_002280644.4 Replaced by: Gene Id: 100268056; LOC 100268056: 17.3 kDa class II heat shock protein *V. vinifera* (grapevine)	F: CGTCAAGGAGTACCCCAATTC R: AACTTCCCCACCCTCCTCT	177	[23]

* NIH—NCBI: National Library of Medicine—National Center for Biotechnology Information.

**Table 2:** Gene stability ranking of the three candidate reference genes and respective pairwise analyses estimated by different methods. Notes: S.D.—standard deviation; %C.V.—coefficient of variation; *r*—Pearson’s correlation coefficient; M—gene-stability measure; S.E.—standard error.

Candidate Reference Gene	Delta-CT (S.D.)	Bestkeeper	GeNorm (M-Value)	NormFinder (Stability Value ± S.E.)	Comprehensive Ranking (Geomean of S.D. Values)
*PEP*	2.926	S.D. = 2.610 %C.V. = 7.81 *r* = 0.869 (*p* = 0.001)	-	0.117 ± 0.019	0.562
*VAG*	1.126	S.D. = 0.940 %C.V. = 4.13 *r* = 0.336 (*p* = 0.075)	-	0.094 ± 0.018	0.293
*UBC*	0.716	S.D. = 0.520 %C.V. = 2.59 *r* = 0.618 (*p* = 0.001)	-	0.045 ± 0.028	0.227
Pairwise analyses					
*PEP* + *VAG*	2.382	*r* = 0.900	2.698	-	-
*VAG* + *UBC*	0.807	*r* = 0.929	1.391	-	-
*PEP* + *UBC*	2.605	*r* = 0.791	2.723	-	-

**Table 3:** Mean relative expression ratio of the *HSP17.9A* gene (±standard error, S.E.), standard deviation (S.D.), and confidence interval (C.I.) determined per ‘grapevine variety × experimental phase’. For the C.I. determination, an α value of 0.95 was used. Different lowercase letters among mean values represent statistically significant differences (*p* ˂ 0.001). Notes: CTF and CTN—control plants of TF and TN varieties, respectively; Ph1—32 °C for 3 h (heat acclimation); Ph2—42 °C for 1 h (severe HS); Ph3—32 °C for 3 h (first recovery period); and Ph4—24 °C during 24 h (second recovery period).

‘Grapevine Variety × Experimental Phase’	Mean Relative Expression Ratio of the *HSP17.9A* Gene ± S.E.	S.D.	95% C.I.
CTF	0.995 ± 0.095 a	0.136	0.99–1.00
CTN	1.085 ± 0.065 a	0.086	1.08–1.09
Ph1_TF	1.345 ± 0.135 a	0.190	1.34–1.36
Ph1_TN	4.310 ± 0.280 b	0.399	4.29–4.33
Ph2_TF	43.815 ± 5.995 d	8.479	43.34–44.29
Ph2_TN	185.405 ± 3.485 e	4.923	185.13–185.68
Ph3_TF	54.265 ± 2.545 d	3.600	54.07–54.47
Ph3_TN	9.590 ± 0.240 b	0.339	9.58–9.61
Ph4_TF	6.280 ± 0.080 b	0.111	6.27–6.29
Ph4_TN	18.110 ± 0.170 c	0.240	18.10–18.13

## Data Availability

The raw data supporting the conclusions of this article will be made available by the authors on request.

## Supplementary Materials

The following supporting information can be downloaded at https://www.mdpi.com/article/10.3390/genes15101283/s1, Figure S1: (**a**) Mean Cq (±standard deviation) values resulting from two biological and technical replicates (n = 2) of the two selected reference (*VAG* and *UBC*) and target (*HSP17.9A*) genes; and respective (**b**) normalised mean ΔCq (±standard deviation) values that were used for the calculation of the relative expression ratio of the *HSP17.9A* gene for each ‘grapevine variety × experimental phase’ interaction. Note: TF—“Touriga Franca”; TN—“Touriga Nacional”; Ph1—heat acclimation (32 °C—3 h); Ph2—severe HS (42 °C—1 h); Ph3—first recovery period (32 °C—3 h); and Ph4—second recovery period (24 °C—24 h).
