# Supplementary material for: Selection of Reference Genes and HSP17.9A Expression Profiling in Heat-Stressed Grapevine Varieties
Source: Genes (Basel). 2024 Sep 30;15(10):1283. doi: 10.3390/genes15101283 (PMC11507026; doi:10.3390/genes15101283)
Supplement: Supplementary file 1 [file genes-15-01283-s001.zip › Figure S1.pdf]

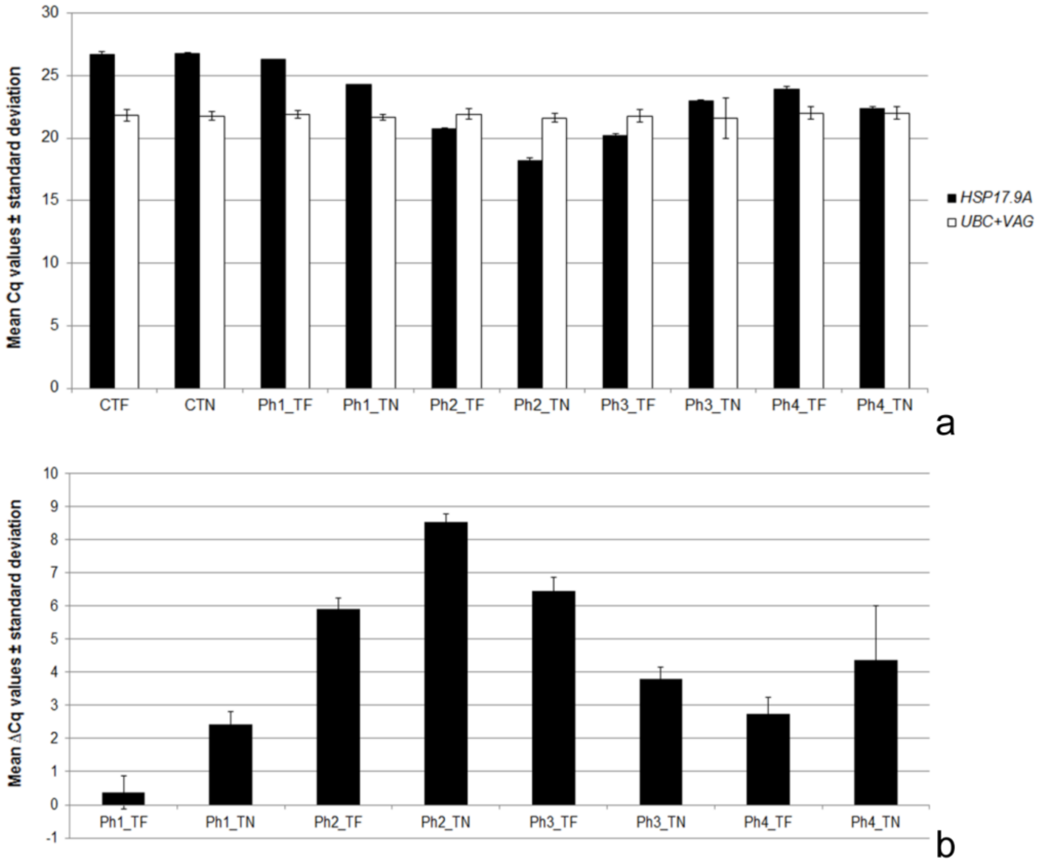

Figure S1. (a) Mean Cq (±standard deviation) values resulting from two biological and technical replicates (n = 2) of the two selected reference (*VAG* and *UBC*) and target (*HSP17.9A*) genes; and respective (b) normalised mean ΔCq (±standard deviation) values that were used for the calculation of the relative expression ratio of the *HSP17.9A* gene for each factors 'grapevine variety × experimental phase' interaction. Note: TF—"Touriga Franca"; TN—"Touriga Nacional"; Ph1—heat acclimation (32 °C—3 h); Ph2—severe HS (42 °C—1 h); Ph3—first recovery period (32 °C—3 h); and Ph4—second recovery period (24 °C—24 h).
